# DEVELOPING A SCALE TO MEASURE NEGLECT SEVERITY: THE HEALTH-RELATED SEVERITY IN ELDER NEGLECT SCALE

**DOI:** 10.1093/geroni/igad104.0453

**Published:** 2023-12-21

**Authors:** Tony Rosen, Karl Pillemer, David Burnes, Terry Fulmer, Jeanne Teresi, Monika Safford, Sara Czaja, Mark Lachs

**Affiliations:** Weill Cornell Medicine, New York City, New York, United States; Cornell University, Ithaca, New York, United States; University of Toronto, Toronto, Ontario, Canada; The John A. Hartford Foundation, New York City, New York, United States; Columbia University Stroud Center, New York City, New York, United States; Weill Cornell Medical College, New York City, New York, United States; Weill Cornell Medicine, New York City, New York, United States; Weill Cornell Medicine, New York City, New York, United States

## Abstract

Caregiver neglect in older persons can vary dramatically in severity, with differential impact on an older adult’s health. Assessing severity is critical for research and clinical practice but has received focus until recently. To address this gap, we developed a scale to describe the health-related severity of elder neglect using an expert consensus method. In development, the experts conceptualized severity as: (1) the level of risk that neglectful behaviors would cause morbidity or mortality and (2) related timeframe. Additionally, the experts recommended that the scale identify risk for future neglect. The scale was designed iteratively, and, after finalization, we assessed face and construct validity. The final scale was found to have validity. It has 5 levels: not present, not present / potential risk, present / mild, present / moderate, present / severe. Each level has a description to guide assessment. For example, present / mild is described as: “caregiving behaviors not optimal, with potential to create morbidity, but low concern for immediate danger,” present / moderate is: “caregiving behaviors with significant potential to create morbidity within the next 4 weeks,” and present / severe is “caregiving behaviors creating immediate danger of morbidity or mortality -- insufficient access to shelter, food, medication – with alternative living situation or ED visit / hospitalization recommended.” The description of not present / potential risk is: “though neglect not currently occurring, factors present that raise concern for future neglect risk.” Assessing neglect severity using this scale may improve understanding of the phenomenon and inform intervention.

